# Integrative and mind-body approaches to law enforcement professional wellness

**DOI:** 10.3389/fpubh.2026.1900573

**Published:** 2026-08-26

**Authors:** Julie Eitzen, Stefanie Holden, Lauray MacElhern, Cassandra Vieten

**Affiliations:** Centers for Integrative Health, Department of Family Medicine, University of California, San Diego, San Diego, CA, United States

**Keywords:** exercise, health, law enforcement, mindfulness, nutrition, police, stress, wellness

## Abstract

**Background:**

Law enforcement (LE) personnel have one of the most stressful jobs, and LE in the United States face a particular set of stressors that merit investigation. LE personnel regularly encounter some of the worst aspects of human nature, which elevates their risk for mental and physical health problems. This population needs viable, accessible methods for stress management and healthy lifestyle education. Integrative and mind-body practices represent one such solution. This structured review aims to assess the effectiveness and promise of integrative health interventions studied in US LE, provide researchers with a resource for conducting future studies, and draw attention to the need for wellness resources for LE personnel in the US.

**Methods:**

PubMed was searched for studies published in 2000 or later that examined an integrative or mind-body health intervention with US LE personnel, including yoga, mindfulness, diet, exercise, or any combination thereof. We reviewed the measures, outcomes of interest, and intervention methodology used across studies.

**Results:**

We identified 21 eligible studies encompassing six major intervention categories: mindfulness, self-regulation, wellness programs, sleep, yoga, and detoxification approaches. Outcomes of interest fell into 10 categories: stress and burnout, mental health, physiological and physical, anger and aggression, job satisfaction and performance, sleep, positive psychology, cognitive, health behaviors, and social. We also compiled the measures used across studies into two overarching categories: survey and qualitative measures, and physical/physiological measures (e.g., sleep duration, strength, BMI).

**Conclusions:**

The results of this review can be used to inform future studies on this population and help formulate wellness programs for LE. Mindfulness interventions generally improved mental health, burnout, mindfulness, and organizational stress; the single study measuring aggression found a significant improvement. Self-regulation interventions improved some physical/physiological outcomes but not others, with no change in sadness, anger, or anxiety. Wellness program interventions showed promise for improving health behaviors, particularly eating habits, tobacco use, strength, and avoidance coping. Yoga was beneficial for global mood and perceived stress. Results were mixed both between and within studies of the same intervention type. Future research with this population should focus on determining the most efficient and effective interventions for US LE personnel.

## 1 Introduction

Police work is one of the most stressful professions. Repeated exposure to traumatic critical incidents (1, 2), occupational stressors (shift-work), frequent involvement in dangerous situations, frequently witnessing pain and suffering of others, encountering the darkest side of human behavior, and criticism from the public and press contribute to this already challenging occupation. Unsurprisingly, police personnel are at higher risk for developing mental and physical health difficulties (2–5), including trauma-related psychological disorders (3, 6); depression and anxiety (4); sleep issues (6, 7); musculoskeletal problems (8, 9); and metabolic and cardiovascular-related diseases (10–12). Those in LE professions are more likely to die by suicide than in the line of duty (10) and more likely to die by suicide than those in most other occupations (13). United States LE personnel face a unique set of stressors that may incur a particular set of harmful effects. US LE training is shorter than some other democratic countries, US police training can lack centralization or standardization, and US LE personnel hold more responsibilities (crimes, emergency responses, traffic citations) than other countries (14). Current media coverage of use of force among US LEOs adds a compounding layer to this. Increasing recruitment difficulties (15) and understaffing (3) further impacts overworked LE personnel.

The nature of LE work is inherently stressful; this is unlikely to change. However, we can do a better job of providing preventative resources and training at the organizational and individual levels to both reduce and build resilience to trauma and stress. It is vital that those in the LE profession be provided with effective tools and techniques to manage stress in order to cultivate protective measures against possible physical and mental health problems. LEs often do not seek help until secondary effects of stress are manifesting, such as relationship or marriage difficulties, panic attacks, or symptoms of PTSD, depression, or anxiety (16). Protective factors may mitigate the development of mental health problems and psychological disorders. For example, trait-based resilience may provide some protection from PTSD symptoms for LE personnel (17). However, in general populations *learned* resilience, or training people to modulate their reaction to the amount or intensity of perceived stress in one's life, can be an inexpensive, effective way to protect against stress and mental health problems (18). Mindfulness training, often included in mind-body interventions, has demonstrated improvement in managing cardiovascular disease symptoms and psychological outcomes within the general population (19, 20). Mindfulness training has consistently shown improvement in PTSD symptoms, including in veterans (21, 22). Learning how stress resilience can be trained in law enforcement professionals could have profound benefits.

Because stress affects brain function as well as several physiological systems including the cardiovascular, musculoskeletal, gastrointestinal, and the nervous system (23), the mental and physical health of LE personnel may benefit from psychopsychological or mind-body resilience training. Mind-body, integrative or whole-person health practices, demonstrate promising outcomes in treating or alleviating many of the problems sworn and non-sworn police personnel experience as the result of working an exceptionally stressful, emotionally and physically taxing job (24). Integrative approaches are characterized by a whole-person orientation: rather than targeting a single symptom or diagnosis in isolation, they combine complementary strategies, such as mindfulness, movement, nutrition, and stress-management training, to address physical, psychological, and behavioral health together. This orientation is particularly relevant to LE personnel, who frequently present with a constellation of co-occurring symptoms (e.g., disrupted sleep, elevated cardiovascular risk, anxiety, and burnout) rather than a single isolated problem, and who may therefore benefit more from multicomponent approaches capable of addressing several of these symptoms simultaneously than from interventions that target only one outcome at a time.

### 1.1 Aim

In this structured review (a review that poses a specific research question with a well-defined scope), our aim was to assess current knowledge on the effects of integrative health or mind-body interventions on health outcomes of law enforcement personnel in the United States. We focused specifically on the US context because, as noted above, US LE training, staffing, structures, and scope of duties differ meaningfully from those in other democratic countries (14), such that findings from international LE samples may not generalize directly to the US workforce, in order to inform a program being created for California law enforcement agencies. We reviewed studies that focused on multicomponent or comprehensive wellness programs (e.g. addressing a combination of nutrition, exercise, mindfulness, and sleep at once, rather than being targeted toward one outcome) that were directed toward alleviating stress-related mental health issues, improving physical health markers, increasing holistic wellness, or generally managing stress.

## 2 Method

For this review, we were specifically interested in studies from the year 2000 and onward with the US law enforcement or police personnel (sworn and unsworn) population. Any studies that used the term “first responders” or terms similar to that and did not identify LE as a subpopulation in the study were excluded. Only articles written in English were included. We chose the year 2000 as a starting point because integrative and mind-body interventions were rarely studied in occupational or LE-specific samples prior to this period, and because LE training, staffing, and organizational practices in the US have changed substantially over the past two and a half decades, such that earlier findings may have limited relevance to the current occupational context.

We included studies that used evidence-based mind-body, integrative health and wellness interventions that have been correlated with improved physical and mental health and wellness. Studies with or without control or comparison groups were included. Studies were included that measured any physical or mental wellbeing or health outcome, or bio-psycho-social-spiritual outcomes. Consistent with a PICOS framework, eligible studies were required to sample US LE personnel (Population); deliver a mind-body, integrative, or whole-person wellness intervention, whether single-component or multicomponent (Intervention); compare to a no-treatment, waitlist, or active comparison group where available, though a comparator was not required for inclusion (Comparator); and report at least one quantitative or qualitative physical, mental, or bio-psycho-social-spiritual health outcome (Outcome). We did not restrict inclusion by study design (Study design): eligible designs ranged from randomized controlled trials to quasi-experimental, single-arm pre-post, and feasibility/pilot studies. This broad design criterion reflects the current state of the literature in this population, in which controlled and randomized designs remain uncommon; the resulting heterogeneity in methodological rigor across included studies is addressed further in the Discussion (see Limitations).

### 2.1 Search strategy

The PI (CV) and one other researcher (JE) drafted a protocol for inclusion/exclusion criteria based on our aim (to review comprehensive integrative mind-body programs directed at multiple health outcomes in LE settings). Based on this, a search string was developed by one researcher and a reference librarian skilled in literature search. We searched the PubMed database with the following search string (note: mesh restricts searches to articles specifically indexed with a topic; tiab = title and abstract; mh = articles which contain the term as either a minor concept or as major concept):

((Police[Mesh] OR police officer^*^[tiab] OR peace officer^*^[tiab] OR law enforcement[tiab]) AND (integrative[tiab] OR holistic[tiab] OR mind-body[tiab] OR “whole health” OR “mind body spirit” OR biopsychosocial[tiab] OR wellbeing[tiab] OR wellness[tiab] OR “physical health”[tiab:~1] OR mental health OR “stress management”[tiab:~1] OR “Holistic Health”[Mesh] OR “Integrative Medicine”[Mesh] OR “Mind-Body Therapies”[Mesh] OR “Mental Health”[Mesh] OR “Mindfulness”[Mesh] OR mindfulness[tiab])) AND (reduce^*^ OR improv^*^ OR impact^*^ OR measur^*^ OR mitigate^*^ OR outcome^*^ OR efficacy OR benefi^*^ OR Treatment Outcome[mh] OR Program Evaluation^*^[mh] OR “no change” OR effective^*^ OR insignificant OR significant).

The results from this search were exported to a spreadsheet and the articles were screened by one researcher (JE). Articles were first included or excluded based on title and abstract with regard to the inclusion/exclusion criteria described above. If unclear, the remaining articles were included or excluded based on the full text of the article. We searched PubMed alone because it is the primary indexing database for peer-reviewed biomedical and health literature and offered adequate coverage for this review's aim of characterizing published, peer-reviewed evidence on integrative interventions in US LE populations; we did not search gray literature sources (e.g., conference proceedings, unpublished reports, agency white papers), which may have excluded relevant applied or field-based work. We revisit this as a limitation of the review below (see Discussion).

## 3 Results

The modified PRISMA (25) diagram in Supplementary Figure 1 describes the study identification, screening, and inclusion process. Of 1,364 original articles, 1,284 were excluded based on title and abstract. This left 80 remaining articles. The full text of these 80 articles were screened for inclusion/exclusion, resulting in 17 studies that met our eligibility criteria from our search string. Two more studies were found in review articles that appeared in the PubMed search, and were added to our review. These two studies did not show up in the original search of the database. One article (26) was found in a systematic review (27). The other study was a dissertation (28) and was extracted from a meta-analysis (29). Lastly, two additional studies meeting our inclusion criteria (30, 31) were referenced by others and included in this review. This brought the total number of studies reviewed to 21.

Below we summarize what the main findings of each study were, the types of interventions employed, what measures were used, and effects of interventions on outcomes.

### 3.1 Results by intervention

We grouped the main categories of intervention methods into mindfulness, self-regulation, general wellness, yoga, detoxification, and sleep (see Table 1 and Supplementary Table 1). There were nine mindfulness-based studies (26, 32–39), six self-regulation studies (28, 40–44), two general wellness program studies (30, 45), two yoga studies (31, 46), one detoxification study (47), and one sleep study (48).

**Table 1:** Mind-body and integrative health intervention categories identified across 21 studies of US law enforcement personnel.

Intervention category	Studies	Definition/core components
Mindfulness-based	9	8-week (one 5-day), 18–20-h, police-specific programs adapted from Mindfulness-Based Stress Reduction and Mindfulness-based resilience training frameworks; formal meditation, body-scan, and breath-awareness practice.
		One variant embedded mindfulness within imagery-based rehearsal of stressful scenarios, led by senior officers rather than mental health professionals.
Self-regulation	6	Biofeedback/heart-rate-variability (HRV) training (e.g., HeartMath coherence programs) teaching breathing and physiological self-regulation skills, ranging from a single-session smartwatch stress-alert protocol to 16-week programs combining device-assisted HRV coherence training with education and mentoring.
		One variant adapted stress inoculation training (a CBT-based approach) with mind-body relaxation techniques.
General wellness program	2	Multi-component, 10-week to 6-month programs combining nutrition, exercise, peer/team support, sleep, and stress-management coaching or seminars.
Yoga	2	6 to 20-week web-based or in-person yoga programs (e.g., YogaShield, Kripalu yoga) combining postures, breathing exercises, meditation, and psychoeducation.
Sleep	1	12-week sleep-hygiene and education program adapted from a NIOSH shift-work curriculum, including a sleep diary and actigraphy monitoring.
Detoxification	1	4 to 6-week sauna-based detoxification protocol with nutritional supplementation and exercise, targeting LE personnel with occupational methamphetamine exposure.

Some interventions combined elements across categories.

### 3.2 Outcome measurement

We grouped measurement methods across studies into self-report measures and physical/physiological measures (see Supplementary Table 2). Self-report measures included psychological and physical factors measured via surveys and questionnaires examining mental health, affect, stress and burnout, sleep, physical health, work limitations, etc. Physical/physiological measures included items such as blood pressure, BMI, cholesterol levels, cortisol levels, glucose levels, heart rate, heart rate variability, height, inflammatory markers, sleep measures, and weight.

Just over half of the studies included both self-report and physical measures to gauge outcomes such as stress, burnout, resilience, mental health, and health behaviors. While there was only one sleep intervention, seven studies measured sleep outcomes (32–34, 38, 44, 45, 48). The mindfulness and self-regulation studies generally used a more comprehensive set of measures (survey/qualitative and physical/physiological tests and measures). The general wellness intervention study (45) also included a broad spread of measures including those focused on health behaviors, substance abuse, sleep, mental health, and physiology.

### 3.3 Results by outcome

Table 2 and Supplementary Table 3 contains the same data as Table 1 and Supplementary Table 1, but summarizes results of each intervention categorized by outcome, indicating which outcomes showed improvements, worsening, or no change. Some articles are repeated because they measure multiple types of outcomes. The main categories of outcomes of interest found in this review were: stress and burnout, mental health, anger and aggression, job satisfaction and performance, sleep, positive psychology, cognitive, health behaviors, and social support as well as physical/physiological.

**Table 2:** Outcome categories assessed across studies of mind-body and integrative interventions in US law enforcement personnel.

Outcome category	Studies (k)	Representative measures
Stress and burnout	16	Perceived stress scale; operational/organizational police stress questionnaire; Maslach and Oldenburg burnout inventories
Mental health	15	PTSD checklist (DSM-5/Civilian); PROMIS anxiety and depression short forms; hospital anxiety and depression scale; suicidal ideation (concise health risk tracking scale)
Physiological/physical	10	Salivary and hair cortisol; blood pressure; heart rate variability; BMI; inflammatory markers (CRP); lipid panel/HbA1c
Job satisfaction and performance	9	Job satisfaction measure (Pond and Geyer); work limitations questionnaire; health and work performance questionnaire
Anger and aggression	9	Buss-perry aggression questionnaire-short form; PROMIS anger short form
Sleep	8	Pittsburgh sleep quality index; actigraphy; epworth sleepiness scale; sleep diaries
Positive psychology	7	Five facet mindfulness questionnaire; connor-davidson/brief resilience scales; self-compassion scale; brief COPE
Cognitive	6	Mini-mental state examination
Health behaviors	6	Alcohol use disorders identification test; health behaviors checklist; fruit/vegetable consumption screener; tobacco use
Social	6	Family assessment device; sources of support scale; PROMIS social functioning

Some interventions combined elements across categories.

#### 3.3.1 Stress and burnout

Sixteen studies examined stress and burnout outcomes. This included burnout, general stress, fatigue, and types of stress (organizational, operational, perceived, etc.; note that while cortisol levels are strongly associated with stress and burnout, we list cortisol under the physical/physiological section). All but one of the studies that measured stress and/or burnout demonstrated improvement in these outcomes: two of those 15 studies were secondary analyses of another included study (38). Three (40, 42, 44) of the 15 studies showed qualitative improvement with one (40) of those three being a purely qualitative study. Another two studies showing qualitative improvements (42, 44) found no change in quantitative outcomes.

Grupe and colleagues (34) used a principal components analysis to create composite outcome variables, showing strong improvement in a composite outcome they labeled “Distress.” This factor included the Perceived Stress Scale; PTSD Checklist; PROMIS anxiety, depression, fatigue, and social participation; the Work Limitations Questionnaire; and the Oldenburg Burnout Inventory (OLBI). While we have included this factor in the Stress and Burnout category, it has relevance for mental health as well.

Some measures of stress and burnout did not change in response to intervention: one study of the 15 found trend level improvements (30). This was reported as a significant reduction in stress in response to the intervention. However, the *p*-value of 0.058 indicates this result may have been better interpreted as a trend.

One study found a dose-response relationship between degree of intervention engagement and *increased* occupational stress (34). While non-significant, Ramey and colleagues (42) reported that one measure was “opposite to improvement” (p. 799), specifically “relational tension” (*d* = 0.21), a subscale of the Organizational Stress scale on the Personal and Organizational Quality Assessment.

We included the Intention to Quit subscale of the Organizational Stress primary scale on the Personal and Organizational Quality Assessment (POQA) here in Stress and Burnout as well as Job Satisfaction and Performance. No change was found in studies that included this subscale.

Grupe and colleagues (34) found significant improvement over time in the Operational subscale of the Police Stress Questionnaire. The Organizational subscale trended toward improvement from T2–T1 (*p* = 0.054) and remained significantly improved at T3–T1 (*p* < 0.0005; note that these results are not included in the published article, but were obtained via personal communication).

Of the 15 studies that showed improvement, six were self-regulation interventions (28, 40–44), six were mindfulness (26, 33–36, 38), with two of these being secondary analyses (35, 36), two were wellness programs (30, 45), though one of these showed trend level improvement (30), and two were yoga interventions (31, 46).

#### 3.3.2 Mental health

Fifteen studies included mental health results. Outcomes in this domain ranged from anxiety, depression, PTSD symptoms, and emotional or affective tendencies. Of the 10 studies that demonstrated improvements, five of these were mindfulness-based interventions, two were self-regulation focused, two involved yoga (one of those showed trend improvements), and one detoxification. Improvements were found in 10 studies and included mood and emotional states, anxiety, depression, and PTSD symptoms.

Of the five studies that evaluated PTSD symptoms, four showed improvements, one of which (32) was a secondary analysis of the Grupe and colleagues' (34) study. Another of those four studies found non-significant but trending improvement (31). While non-significant, Ramey and colleagues (42) reported that two subscales of a post-traumatic stress measure yielded results that were “opposite to improvement” (p. 799): the Intrusive (*d* = 0.26) and Avoidance (*d* = 0.29) subscales of the Impact of Events Scale. No studies reported mental health outcomes that significantly worsened.

Grupe and colleagues (34) found improvement in anxiety at the post-training assessment, 3 month follow up testing, and over time. PTSD symptoms improved at all testing times except at 3 months, and depression showed improvement only at 3 months. The composite outcome of Distress (which is also under our Stress and Burnout category as its constituent components relate to both these categories) showed improvement over time and at both testing times.

There were trend level improvements in two studies. These were found in PTSD symptoms (*p* = 0.05) in Tan et al.'s (31) study, depression (*p* = 0.06) in Grupe et al.'s (34) study, and anger/hostility (*p* = 0.07) in Jeter et al.'s (46) study. Some studies found no improvement in any mental health measures (38, 41, 42).

#### 3.3.3 Physiological and physical

Ten studies measured physical/physiological outcomes. Self-report measures included general health, energy levels, and physical symptom severity. Physiological measures included lab work on cortisol levels, cholesterol levels, blood glucose, and inflammatory markers. Other physical measures included heart rate, body weight, strength, heart rate variability, coherence, and blood pressure. Eight of these studies found improvements. Of these eight, five also found outcomes that remained the same.

Two (34, 38) of the three studies that measured cortisol found improvement and significant interactions. Christopher and colleagues (38) found significant improvement in cortisol levels in the treatment group on the third day after post-training and significant group-by-time (and, specifically to this study, group-by-time by day-by-minute) interaction, but no improvement at 3 months. A separate study by Christopher and colleagues (26) found no improvement in cortisol levels, but there were trend level improvements in pain interference (*p* = 0.07) and significant improvement in self-reported physical health.

McCraty and Atkinson (44) recorded greater increased heartbeats after intervention on a self-report measure, which is difficult to interpret. Additionally, two studies did note that, while not statistically significant, some effect sizes indicated worsening. This was the case for LDL cholesterol (*d* = 0.27) in Ramey and colleagues' (42) study. In Grupe and colleagues' (34) study, pain interference (T2–T1 *d* = 0.35; T3–T1 *d* = 0.42), pain intensity (T2–T1 *d* = 0.30; T3–T1 *d* = 0.38), and physical functioning (T2–T1 *d* = 0.10; T3–T1 *d* = 0.33) were also non-significant findings that indicated numeric worsening. While the detoxification study (47) found 18 physiological measures that improved, this study used a particular subgroup of LEOs that were exposed to methamphetamine sites and results may not apply to other LEOs. The detoxification study also provided a list of symptoms that occurred during the sauna treatment. This list is included in Supplementary Materials Table 1 under the Qualitative column. No statistical analyses were performed on these particular findings, they were provided by the authors for transparency and completeness.

#### 3.3.4 Anger and aggression

Nine studies measured anger and aggression outcomes: one of these studies (37) was a secondary analysis of the 2018 study by Christopher and colleagues (38). Of these eight studies, four found improvement in anger or aggression. One of these four studies that found improvement (46) was not significant but did find trending improvement (*p* = 0.07) in the Anger/Hostility subscale of the Profile of Mood States Short Form (POMS-SF) measure. Of the eight studies that measured anger and/or aggression, four were self-regulation interventions, three were mindfulness interventions (with one of those three being a secondary analysis), and one was a yoga-based intervention. No studies found any worsening of anger or aggression.

#### 3.3.5 Job satisfaction and performance

Nine studies examined workplace outcomes. These included job satisfaction, work performance, productivity, absences, intention to quit, and physical or psychological problems that impede work roles. Two (44, 47) of these nine studies showed improvement in this category of outcomes. McCraty and Atkinson (44) found improvements in work performance while Ross and Sternquist (47) found improvements in role limitations due to physical health or emotional problems, and days of limited activity due to poor health or sick days, but no change in productivity.

Two studies (31, 34) that measured health-related productivity loss found no improvement in this particular outcome. Two studies (30, 44) measured job satisfaction and both of those studies found no change in the measure. Three studies that measured Intention to Quit (which is also included in our Stress and Burnout section) found no changes in that outcome (41–43). No studies found any worsening outcomes.

While both studies that measured aggression found improvements (37, 38), Ribeiro and colleagues' study was a secondary analysis of Christopher and colleagues' work. Only two studies out of the seven that measured anger found improvement (26) with one of those studies showing non-significant but trending levels of improvement (46).

#### 3.3.6 Sleep

Eight studies evaluated sleep outcomes: one of these studies (32) was a secondary analysis of Grupe and colleagues (34). Outcomes measured included sleepiness, sleep disturbances, sleep latency, subjective and objective measures of sleep quality, and nightmares among other measures (please see Supplementary Table 3). Five studies (32–34, 38, 39) used mindfulness-based interventions (including 32, which was a secondary analysis). One was a self-regulation study (44), one a wellness program intervention (45), and one was a sleep study, specifically (48). Six studies found improvement in sleep outcomes (including the secondary analysis) and five of those studies also found outcomes that did not change. The secondary analysis (32) found that improvement in subjective sleep quality was mediated by reductions in PTSD symptoms. No studies found any worsening outcomes for sleep.

#### 3.3.7 Positive psychology

Seven studies included positive psychology measures. These outcomes ranged from mindfulness, dimensions of mindfulness, psychological flexibility, self-compassion, resilience, and coping. Six of these studies found improvements in these areas, however, one study was a secondary analysis of Christopher and colleagues' 2018 study (36, 38). Of the six studies that found improvement, three also found some aspects of positive psychology factors that showed no change. Trend level improvements were found in the Jeter and colleagues (46) study and McCraty and Atkinson's (44) study. Jeter and colleagues found trend level improvements in the Observing subscale of the mindfulness measure (*p* = 0.10). McCraty and Atkinson reported a *p-*value of <0.056 for Vitality and peacefulness (*p* < 0.056) in McCraty and Atkinson's (44) study, which they labeled as “marginally significant” (p. 53; it should be noted, however, the *p*-value from McCraty and Atkinson from the provided table is different from the value printed in the Results section of the paper. The Results section *p*-value reads as *p* < 0.56, which the authors acknowledged was in error via personal communication). In addition, the terms “Vitality” and “Peacefulness” were not defined in the article. While the researchers reported using the Personal and Organizational Quality Assessment (POQA) measure, the primary scales and subscales appeared to differ from other reports using the POQA.

No studies found any worsening of positive psychology outcomes but other results were mixed. The Non-judging and Acting with Awareness subscales of the mindfulness measure and the overall score for the mindfulness scale were found to have no change in the Christopher and colleagues' (38) study while Non-reactivity showed improvement. In a separate study by Christopher and colleagues (26), however, improvement was found in the overall score and all subscales of the mindfulness measure used.

#### 3.3.8 Cognitive

Six studies analyzed cognitive outcomes. These outcome measures included coping strategies, cognitive effectiveness, cognitive function, emotional intelligence, goal clarity, and mental clarity. Four of these six studies found improvement in cognitive outcomes. Existing negative cognitive symptoms improved for the methamphetamine exposure detoxification study (47) and emotional intelligence showed improvement in Christopher and colleagues' (26) study. Coping strategies results were heterogeneous. Across the studies, outcomes of humor, positive reframing, and avoidance coping strategies showed improvement whereas effective coping, positive planning, self-blame, coping skills, and approach coping strategies showed no change. Cognitive effectiveness also showed no change. No cognitive measure resulted in worsening outcomes after interventions.

#### 3.3.9 Health behaviors

Six studies examined health behavior outcomes. Measures included alcohol use, tobacco use, fruit and vegetable consumption, healthy eating, and physical activity. Of these six studies, five found some improvement in health behavior outcomes. Two of these five studies were secondary analyses of Christopher and colleague's 2018 study (38). Rehder and colleagues (35) found that reduced alcohol consumption was mediated by reduced burnout and Kaplan and colleagues (36) found that reduced alcohol use was predicted by increased mindfulness. While results varied depending on the testing schedule (please see Supplementary Table 3), Kuehl and colleagues (45) found improvement in fruit consumption, vegetable consumption, fruit and vegetable consumption, healthy eating, and tobacco use when comparing the average of all follow-up measures to baseline. Of the three primary studies (e.g., not secondary analyses) that measured alcohol use, all three showed improvements during some point in testing. Kuehl and colleagues (45) found improvement in alcohol use in the 12 month follow-up only while Christopher and fellow researchers (38) found a significant interaction over time but at post-analyses, no improvements were found. No change was found with follow-up measures, either. No health behavior measures showed worsening outcomes.

#### 3.3.10 Social

Six studies looked at social outcomes and two of these found improvement while the rest found no changes. One of those two studies found only qualitative results improved but no quantitative outcomes improved (44). Main measures and subscales included family relationships, interpersonal skills, communication, social support and participation. Social functioning showed improvement in Ross and Sternquist's 2011 methamphetamine exposure detoxification study (47). All four of the studies with no changes were mindfulness-based interventions. The study that found improvement in qualitative results but not quantitative was a self-regulation intervention (44). While no studies found statistically significant worsening of outcomes, Grupe and colleagues (33) found that the effect sizes of social participation showed numeric worsening (T2–T1 *d* = 0.28; T3–T1 *d* = 0.22).

## 4 Discussion

The findings of this structured review reveal a compelling narrative about the potential of integrative health interventions to address the complex wellness challenges facing law enforcement personnel. Rather than simply cataloging positive outcomes, these results illuminate critical patterns that suggest a fundamental shift may be needed in how we approach officer wellbeing and organizational health within law enforcement agencies. We note at the outset that most included studies used non-randomized, single-arm, or small pilot designs; the interpretation that follows should therefore be read as identifying promising patterns and hypotheses for future controlled research, not as evidence of causal efficacy.

The consistency of integrative interventions in addressing stress and burnout (15 of 16 studies showing improvement) suggests these approaches may be uniquely suited to the occupational reality of law enforcement. Unlike traditional employee assistance programs that often focus on crisis intervention, mind-body interventions appear to build proactive resilience capacity. This distinction is crucial given that US law enforcement personnel face not only inherent occupational stressors but also contemporary challenges including chronic understaffing, public scrutiny, and organizational instability.

The consistency of stress reduction outcomes across multiple intervention types (mindfulness, self-regulation, wellness programs, and yoga) suggests that the underlying mechanisms, rather than specific techniques, may be driving these improvements. This points to the importance of addressing the psychophysiological stress response system as a whole, rather than targeting isolated symptoms. The robust improvements in positive psychological factors and health behaviors further support this systems-level approach, indicating that successful interventions may be those that enhance overall adaptive capacity rather than merely treating specific deficits.

Perhaps the most intriguing finding is the nuanced pattern of results across different outcome domains. While stress and wellness markers showed consistent improvement, job satisfaction and performance outcomes proved surprisingly resistant to change. This paradox reveals an important tension in occupational health interventions: personal wellbeing and organizational performance may not be as directly linked as commonly assumed.

The mixed results for anger and aggression outcomes, with mindfulness showing promise while self-regulation showed no effect, suggest that not all mind-body approaches are equivalent in their impact on every measured outcome. This finding has significant implications for law enforcement, where emotional regulation capabilities directly impact public safety and community relations. The fact that amount of mindfulness practice was correlated with reduced aggression in one study points to the importance of sustained practice rather than brief exposure to techniques.

The disconnect between improved individual wellbeing but unchanged job performance metrics raises important questions about how we conceptualize and measure success in occupational interventions. It may be that current performance indicators fail to capture the subtler but meaningful changes in officer resilience, decision-making quality, or long-term career sustainability that these interventions provide.

The physiological findings present both encouraging evidence and methodological puzzles that warrant deeper consideration. The delayed cortisol responses observed across multiple studies suggest that stress system recovery may follow a different timeline than subjective symptom improvement. This has profound implications for intervention design and evaluation; brief post-intervention assessments may systematically underestimate physiological benefits.

The superior performance of self-regulation interventions on objective physical measures, contrasted with mindfulness interventions' strength in cortisol regulation, suggests these approaches may target different aspects of the stress response system. This complementarity rather than redundancy in mechanisms points toward the potential value of integrated intervention approaches that combine multiple modalities.

The consistent emphasis on cultural adaptation across successful studies reveals something fundamental about intervention effectiveness in law enforcement populations. The modifications made to standard protocols, such as reducing discussion components, increasing practice frequency while decreasing duration, and involving department personnel in program development, reflect recognition that law enforcement culture includes both barriers (such as reluctance to discuss emotions) and assets (such as discipline and commitment to training) that must be navigated strategically.

The positive participant feedback across intervention types, despite concerns about time commitment, suggests that officers and other law enforcement professionals recognize the value of these approaches when properly adapted. The participant reports of increased stress awareness and improved capacity to handle challenges indicate that these interventions may be developing metacognitive skills that extend beyond the specific techniques taught.

These findings collectively suggest that integrative health interventions represent a viable and valuable approach to law enforcement wellness, but they also reveal critical gaps in our understanding. The underrepresentation of certain intervention types (particularly yoga, sleep, and detoxification approaches) limits our ability to make evidence-based recommendations about optimal intervention selection for specific populations or outcomes. Our search strategy and focus on the US also mean this review does not capture several potentially relevant areas: comparative work on the stressors and interventions used with LE professionals internationally, which could offer useful context even though direct generalization to the US system may be limited given differences in training and organizational structure previously noted; religious and spiritual coping practices, which may serve as a wellness resource not captured by our search terms; and sports, martial arts, or physical training based approaches to stress management, which were outside of our definition of integrative, multicomponent wellness programs but represent a related and growing area of inquiry. We note that each of these represents a promising direction for future reviews and primary research.

The delayed physiological responses and mixed performance outcomes highlight the need for longer-term follow-up studies that can capture the full trajectory of intervention effects. Future research should also prioritize head-to-head comparisons of different intervention types and investigate combined approaches that might leverage the complementary strengths revealed in this review. Perhaps most importantly, the success of culturally adapted interventions points to the need for implementation research that examines how these promising findings can be translated into sustainable organizational practices within law enforcement agencies.

While this review provides valuable insights into the current state of integrative health interventions for law enforcement, several limitations warrant acknowledgment. The reliance on a single database and the inclusion of studies with varying methodological rigor may limit the strength of conclusions that can be drawn. The small number of studies in several intervention categories prevents robust comparison of different approaches. More fundamentally, the field lacks consensus on optimal outcome measures and intervention dosage for this population. The mixed results across studies of the same intervention type suggest that protocol standardization, while maintaining cultural adaptation, may be crucial for advancing the field.

This review reveals that integrative health interventions hold significant promise for addressing the multifaceted wellness challenges facing US law enforcement personnel. However, the pattern of findings suggests that success depends not just on intervention selection but on thoughtful adaptation to law enforcement culture, appropriate outcome measurement, and realistic expectations about the timeline and scope of benefits. Rather than viewing these interventions as simple stress management tools, the evidence points toward their potential role in building comprehensive resilience capacity that can help officers navigate both the acute challenges of their work and the long-term health risks of their profession. The path forward requires continued research that is both methodologically rigorous and practically grounded in the realities of law enforcement work. Only through such an approach can we move beyond promising pilot studies to evidence-based wellness programs that can be sustainably implemented across diverse law enforcement agencies.

## Data Availability

The data analyzed in this study is subject to the following licenses/restrictions: as a structured review, results of prior studies were analyzed, but original datasets were not analyzed. Authors of the published studies reviewed would bear responsibility for whether datasets were made public. Requests to access these datasets should be directed to Cassandra Vieten; cvieten1@health.ucsd.edu.
