# Supplementary material for: Integrative and mind-body approaches to law enforcement professional wellness
Source: Front Public Health. 2026 Aug 26;14:1900573. doi: 10.3389/fpubh.2026.1900573 (PMC13553770; doi:10.3389/fpubh.2026.1900573)
Supplement: Supplementary file 1 [file Supplementary_file_1.pdf]

Supplementary Material

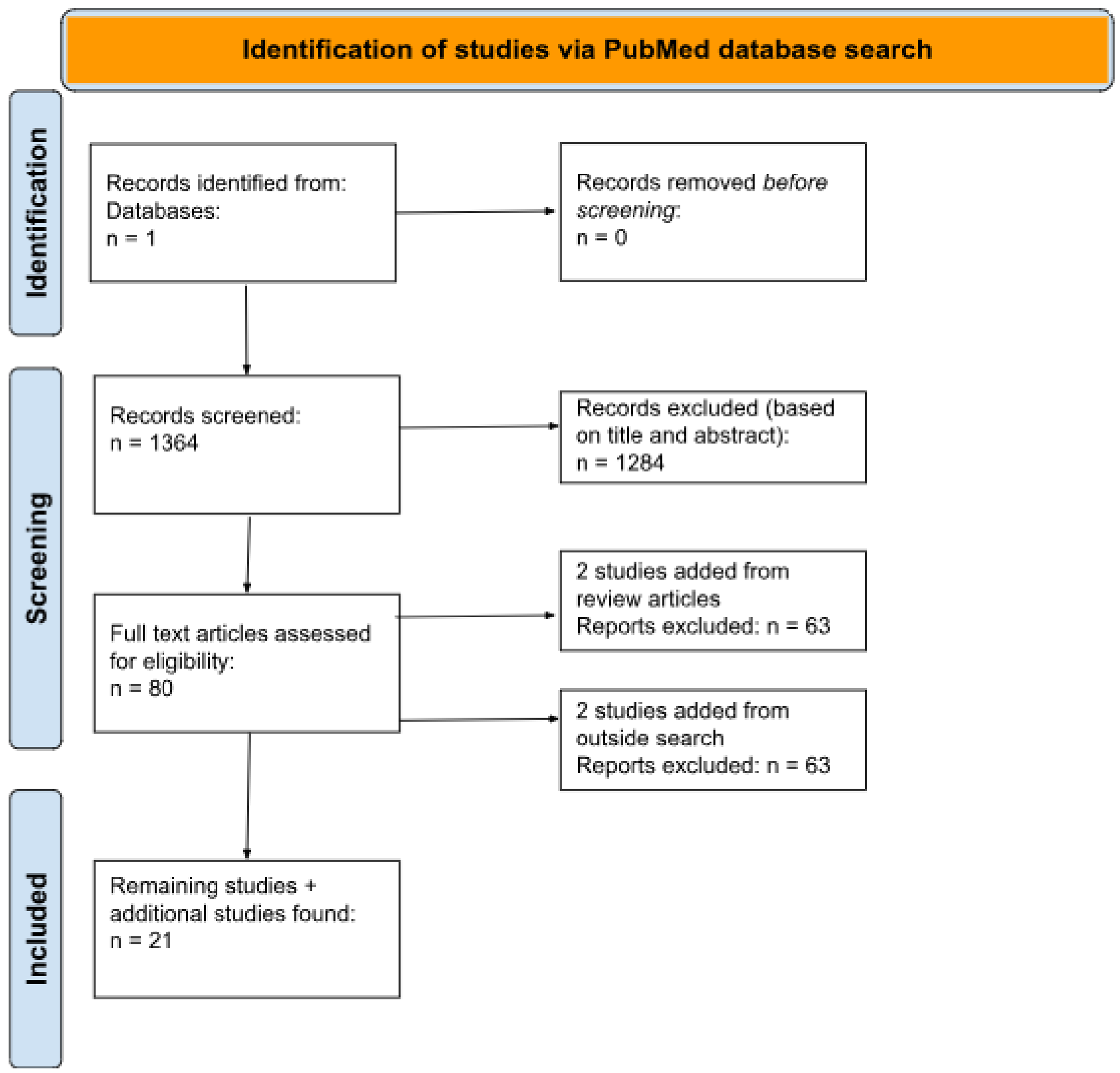

Supplementary Figure 1. PRISMA flow diagram of the study identification and selection process. Records were identified through a search of one database (PubMed) and screened for eligibility; additional studies were identified from the reference lists of review articles and through outside search. A total of 21 studies met inclusion criteria and were included in this review.

Supplementary Table 1: Organized by Intervention Methods

Mindfulness-Based

Study	Intervention description	Improved	Worsened	No Change	Qualitative/Self-Report
32<br><br>Secondary analyses of 34	An 8-week, 18-hour police-specific mindfulness-based training lightly modified from (33,34,49) mindfulness training. inspired by Mindfulness-Based Stress Reduction (MBSR) and Mindfulness-Based Resilience Training (26).	PTSD symptoms<br>Subjective sleep quality (subjective sleep quality improvement was mediated by reductions in PTSD symptoms)	NA	Objective sleep measures (duration & efficiency)	NA
33	An 8-week, 18-hour police-specific mindfulness-based training inspired by Mindfulness-Based Stress Reduction (MBSR) and Mindfulness-Based Resilience Training (26).	PTSD symptoms<br><br>Organizational stress subscale from PSQ<br>T2-T1: $p = 0.054$<br>T3-T1: $p = 0.00042$<br>(Note: Group*time not reported. These values are not included in the published paper—authors provided this information upon request.)<br><br>Operational stress subscale from PSQ<br>T2-T1: $p = 0.03$<br>T3-T1: $p = 0.0016$ (Note: Group*time not reported. These values are not included in the published paper—authors provided this information upon request.)<br><br>Exhaustion subscale of Burnout Measure<br>Anxiety<br>Well-being<br>Negative affect<br>Sleep quality	NA	Burnout (total measure showed no change)<br>Disengagement (subscale)<br>Positive affect<br>Fatigue<br>Sleep disturbances<br>Health-related productivity loss<br>Log-transformed high-sensitivity C-reactive protein<br>Blood pressure<br>Diastolic<br>Systolic<br>Resting pulse rate<br>Social participation<br>NS but effect sizes show numeric worsening (T2-T1 *d* = 0.28; T3-T1 *d* = 0.22)<br>Pain interference<br>NS but effect sizes show numeric worsening (T2-T1 *d* = 0.35; T3-T1 *d* =	NA

Study	Intervention description	Improved	Worsened	No Change	Qualitative/Self-Report
		Depression<br>Trend level improvement ($p = 0.06$)<br>(Note: Authors table indicates $p = 0.067$ whereas results report 0.060)		0.42)<br>Pain intensity<br>NS but effect sizes show numeric worsening (T2-T1 $d = 0.30$; T3-T1 $d = 0.38$)<br>Physical functioning<br>NS but effect sizes show numeric worsening (T2-T1 $d = 0.10$; T3-T1 $d = 0.33$)	
34	An 8-week, 18-hour police-specific mindfulness-based training lightly modified from (33,49) mindfulness training. inspired by Mindfulness-Based Stress Reduction (MBSR) and Mindfulness-Based Resilience Training (26)	**Group*time**<br>Distress Component*<br><br>Sleep Component*<br><br>PTSD Checklist<br>Anxiety<br>Fatigue<br>OLBI Exhaustion<br>PROMIS sleep disturbances<br><br>Cortisol Awakening Response (CAR)<br>Trend level improvement ($p = 0.06$)<br><br>**Post Training**<br>Distress Component*<br><br>PTSD Checklist<br>Anxiety<br>Fatigue<br>OLBI Exhaustion<br>PROMIS sleep disturbances<br><br>**3 Months**<br>Distress Component*<br><br>Sleep Component*	Dose-response for occupational stress<br>(more engagement with practice was associated with significantly **increased** occupational stress)	**Group*time**<br>Pain Component*<br><br>Physical Health Component*<br><br>Occupational Stress Component*<br><br>Perceived stress<br>Depression<br>Social participation<br>Work limitations<br>Pain intensity<br>Pain interference<br>Health behaviors checklist<br>Physical function<br>Operational Stress subscale of PSQ<br>Organizational Stress subscale of PSQ<br>Pittsburgh sleep scale<br><br>Diurnal cortisol slope<br>Hair cortisol concentration<br>Inflammatory markers<br>CRP<br>Cytokines	NA

Study	Intervention description	Improved	Worsened	No Change	Qualitative/Self-Report
		Perceived stress<br>Anxiety<br>Depression<br>Fatigue<br>OLBI Exhaustion<br>Pittsburgh sleep scale<br>PROMIS sleep disturbances<br><br>Cortisol Awakening Response (CAR)<br>Dose response for physical health		**Post Training**<br>Pain Component*<br><br>Physical Health Component*<br><br>Occupational Stress Component*<br><br>Sleep Component*<br><br>Perceived stress<br>Depression<br>Social participation<br>Work limitations<br>Pain intensity<br>Pain interference<br>Health behaviors checklist<br>Physical function<br>Operational Stress subscale of PSQ<br>Organizational Stress subscale of PSQ<br>Pittsburgh sleep scale<br><br>Cortisol Awakening Response (CAR)<br>Diurnal cortisol slope<br>Hair cortisol concentration<br>Inflammatory markers<br>CRP<br>Cytokines<br><br>**3 Months**<br>Pain Component*<br><br>Physical Health Component*	

Study	Intervention description	Improved	Worsened	No Change	Qualitative/Self-Report
				Occupational Stress Component*<br><br>PTSD Checklist<br>Social participation<br>Work limitations<br>Pain intensity<br>Pain interference<br>Health behaviors checklist<br>Physical function<br>Operational Stress subscale of PSQ<br>Organizational Stress subscale of PSQ<br><br>Diurnal cortisol slope<br>Hair cortisol concentration<br>Inflammatory markers<br>CRP<br>Cytokines	
50<br><br>Secondary analysis of 38	An 8-week, 20-hour police-specific Mindfulness-Based Resilience Training (MBRT) that was based on the framework of Mindfulness-Based Stress Reduction	Burnout<br><br>Reductions in alcohol use over time (mediated by reduced burnout)	NA	NA	NA
36<br><br>Secondary analysis of 38	An 8-week, 20-hour police-specific Mindfulness-Based Resilience Training (MBRT) that was based on the framework of Mindfulness-Based Stress Reduction (50).	Burnout<br>Mindfulness<br>Psychological flexibility<br><br>Alcohol use (predicted by increased mindfulness)<br><br>Burnout (predicted by increased self compassion)	NA	Alcohol use<br>Self-compassion<br><br>Neither self compassion nor psychological flexibility predicted decreased alcohol use post treatment<br><br>Neither mindfulness nor psychological flexibility predicted reductions in burnout post treatment	NA

Study	Intervention description	Improved	Worsened	No Change	Qualitative/Self-Report
37<br><br>Secondary analysis of 38	An 8-week, 20-hour police-specific Mindfulness-Based Resilience Training (MBRT) that was based on the framework of Mindfulness-Based Stress Reduction (50).	Aggression<br><br>(Frequency and duration of mindfulness practices was significantly correlated with reduced aggression)	NA	NA	NA
38	An 8-week, 20-hour police-specific Mindfulness-Based Resilience Training (MBRT) that was based on the framework of Mindfulness-Based Stress Reduction (50).	**Group*time**<br>Cortisol (this result was a group-by-time by day-by-minute)<br>Burnout<br>Organizational Stress subscale of PSQ<br>Psychological flexibility<br>Alcohol use<br>Non-reactivity subscale from FFMQ<br>Aggression<br>Sleep disturbance<br>Trend level improvement (p = 0.08)<br><br>**Post Analyses**<br>Cortisol (improvement found on day three post training)<br>Aggression<br>Burnout<br>Organizational Stress subscale of PSQ<br>Non-reactivity subscale from FFMQ<br>Psychological flexibility<br>Sleep disturbance<br>Trend level improvement (p = 0.08)<br><br>**3 Month Follow-up**<br>NA	NA	**Group*time**<br>Anxiety<br>Depression<br>Suicidal ideation<br>Operational Stress subscale of PSQ<br>Non-judging subscale from FFMQ<br>Acting with awareness subscale from FFMQ<br>Resilience<br>Self-compassion<br>Anger<br><br>**Post Analyses**<br>Alcohol use<br>Anger<br>Anxiety<br>Depression<br>Suicidal ideation<br>Operational Stress subscale of PSQ<br>FFMQ<br>Non-judging subscale<br>Acting with Awareness subscale<br>Non-reactivity subscale<br>Resilience<br>Self-compassion<br><br>**3 Month Follow-up**<br>Aggression<br>Anger	NA

Study	Intervention description	Improved	Worsened	No Change	Qualitative/Self-Report
				Alcohol use<br>Anxiety<br>Depression<br>Burnout<br>Suicidal ideation<br>Operational Stress subscale of PSQ<br>Organizational Stress subscale of PSQ<br>FFMQ<br>Non-judging subscale<br>Acting with Awareness subscale<br>Non-reactivity subscale<br>Psychological flexibility<br>Resilience<br>Self-compassion<br>Sleep disturbance	
39<br><br>Participants were police academy trainees	A 5-day, 7.5 hour resilience-focused program that used mindfulness training during imagery of stressful scenarios.<br><br>Imagery rehearsal aspect of intervention based on previous study by (51). Senior officers, rather than mental health professionals, were trained to be leaders in the sessions.	Coping strategies scales from the Brief COPE Scale<br>Positive reframing Humor<br><br>Anxiety<br>Alcohol use	NA	Coping strategies scales from the Brief COPE Scale<br>Active coping<br>Positive planning<br>Self-blame<br>Social support<br><br>PTSD<br>Depression<br>Sleep disruption	NA
26	An 8-week, 20-hour police-specific Mindfulness-Based Resilience Training (MBRT) that was based on the framework of Mindfulness-Based Stress Reduction (50).	Mindful process<br>Mindfulness (overall FFMQ score)<br>Non-reactivity (subscale)<br>Non-judging (subscale)<br>Act with awareness (subscale)<br>Resilience<br>Mental health<br>Physical health<br>Emotional intelligence	NA	Family functioning<br>Salivary cortisol levels	NA

Study	Intervention description	Improved	Worsened	No Change	Qualitative/Self-Report
		Sleep disturbance<br>Anger<br>Fatigue<br>Burnout<br>Difficulties with emotion regulation<br>General stress<br>Organizational Stress subscale from PSQ<br>Operational Stress subscale from PSQ<br>Pain interference<br>Trend level improvement (p = 0.07)			

Self-Regulation

Study	Intervention description	Improved	Worsened	No Change	Qualitative/Self-Report
40	LEOs wore smartwatches for 30 days and received notifications of increased stress when heart rate threshold was exceeded and persisted 10 minutes or longer.<br><br>The wellness intervention was prompted and completed via smartwatch and practiced in the field according to heart rate data.	**Qualitative Data Only**<br>See "Qualitative" column	NA	NA	In interviews, participants reported:<br>• Improved stress awareness<br>• Participants preferred graphic heart rate monitor over stress graphic<br>• Reduction in acute stress from breathing exercises
41<br><br>Participants were police academy recruits	A 6-hour, 2-month program focused on self-regulation techniques to change the stress response via altering breathing and heart rate. Used the HeartMath Inner Balance app and biofeedback.<br><br>Instruction to focus on positive rather than negative emotion.	Coherence (a state of increased synchronization between higher-order brain activity and the two branches of the autonomic nervous system and a shift toward greater parasympathetic dominance)	NA	Perceived stress<br>Vital exhaustion<br>Response to Stressful Experiences<br>Impact of Events Scale<br>Intrusive subscale<br>Avoidance subscale<br>Emotional Vitality<br>Emotional Buoyancy<br>Emotional Contentment<br>Organizational Stress (overall primary scale score)<br>Pressures of Life (subscale)<br>Relational Tension (subscale)<br>Intention to Quit (the job/profession) (subscale)<br>Emotional Stress (overall primary scale score)<br>Anxiety and Depression (subscale)	In anecdotal feedback, participants reported:<br>• Recruits liked the mentoring sessions and requested an additional (fourth) session<br><br>Based on participants' feedback and mental health professional mentors:<br>• It is likely recruits would have gotten more out of the program if it were started earlier in academy training

Study	Intervention description	Improved	Worsened	No Change	Qualitative/Self-Report
				Anger and Resentment (subscale)<br>Physical Stress<br>Fatigue<br>Health Symptoms	
42	A 3-hour, approximately 3-month physiological and psychological resilience intervention.<br><br>LEO received education about stress response and how to alter it, were instructed to engage in independent practice, and had 1 telementoring session.<br><br>The program used HeartMath Coherence Training in biofeedback with self-regulation of breathing, focusing on positive emotions rather than negative to affect physiological stress.	HbA1c<br>Heart rate variability<br>Coherence	NA	Perceived stress<br>Vital exhaustion<br>Response to Stressful Experiences<br>Impact of Events Scale<br>Intrusive subscale<br>NS but authors report effect sizes opposite to improvement (*d* = 0.26)<br>Avoidance subscale<br>NS but authors report effect sizes opposite to improvement (*d* = 0.29)<br><br>Organizational Stress<br>Pressures of Life<br>Relational Tension<br>NS but authors report effect sizes opposite to improvement (*d* = 0.21)<br>Intention to Quit (the job/profession)<br>How Stressed Have You Been<br>Emotional Vitality<br>Emotional Buoyancy<br>Emotional Contentment<br>Emotional Stress<br>Anxiety and Depression<br>Anger and Resentment<br>Physical Stress<br>Fatigue	In a survey at the end of the study, participants reported:<br>- Intervention was valuable<br>- Improved awareness of stress levels<br>- Biofeedback device helped relaxation

Study	Intervention description	Improved	Worsened	No Change	Qualitative/Self-Report
				Health Symptoms<br><br>LDL cholesterol<br>NS but effect sizes opposite to improvement (*d* = 0.27)<br>Blood pressure<br>Cholesterol<br>Triglycerides<br>CRP (inflammatory marker)<br>BMI<br>Waist circumference	
43	After an initial 2-hour training session, a 6-week period of independent work on the Stress Resilience Training System (STRS) administered via iPad app along with meetings with mentors over the phone. Mentor meetings (spread over 4 weeks) lasted 1 hour each. Training sessions consisted of education on stress and used HeartMath self-regulation techniques including HRV coherence with biofeedback.	Emotional Vitality (overall primary scale score)<br>Emotional Contentment (subscale)<br>Physical Stress (overall primary scale score)<br>Health Symptoms (subscale)	NA	Organizational Stress<br>Pressures of Life<br>Relational Tension Stress<br>Intention to Quit (the job/profession)<br>Emotional Stress<br>Anxiety and Depression<br>Anger and Resentment<br><br>Emotional Buoyancy subscale of the Emotional Vitality primary scale of the POQA<br><br>Fatigue subscale of the Physical Stress primary scale of the POQA	In mentor reports based on mentor meetings, participants reported:<br>• Education about coherence and resilience was beneficial to ideal performance<br>• All participants reported that mentors helped in putting new skills into action<br>• Some would prefer shorter mentor sessions; others appreciated longer ones<br>• Overall positive comments from participants about program

Study	Intervention description	Improved	Worsened	No Change	Qualitative/Self-Report
					• Able to apply learned skills into real-life situations, including on-the-job
44	A 16-week, 12-hour intervention that used HeartMath (HM) Coherence Advantage Program which teaches practical and easy-to-execute self-regulation skills and techniques. The experimental group also had access to heart rhythm coherence training devices.<br><br>A subgroup of the experimental group also underwent simulated police call scenarios. 2 scenarios were administered before training and 1 was after training.	Distress<br>Global Negative Emotions<br>Depression<br><br>Work Performance self-ratings from a four-week post-interview of Program Impact Assessment<br><br>Peacefulness<br>trend level improvement ($p < 0.056$)<br>(Note: The publication had *p* values which differed and the value of 0.056 was confirmed by authors via email.)<br>Vitality<br>trend level improvement ($p < 0.056$)<br>(Note: The publication had *p* values which differed and the value of 0.056 was confirmed by authors via email.)	Rapid heart rate (subjective report)	Coping Skills, Family Relationships, and Interpersonal Skills self-ratings from a four-week post-interview of Program Impact Assessment<br><br>Anger<br>Sadness<br>Fatigue<br>Positive Emotion<br>Social Support<br>Mental Clarity<br>Job Satisfaction<br>Goal Clarity<br>Productivity<br>Communication<br>Effectiveness<br>Sleeplessness<br>Anxiety<br>Body Aches<br>Indigestion	In comments during the semi-structured interviews that measured work performance, coping skills, family relationships, and interpersonal skills, the experimental group indicated that, overall:<br><br>• Training was beneficial to managing mood<br><br>• Communication and listening skills were improved<br><br>• Awareness of their stress and emotions improved<br><br>• Training improved participants' capacity to handle stress
28	A 4-week, 6-hour (for the experimental group which also received a booster phone session, it would be approximately 6.5 hours) adaptation of Stress Inoculation Training (SIT) tailored for police.<br><br>This program blends Cognitive Behavioral Therapy with mind-body relaxation and self-regulation	Organizational Stress subscale from the PSQ	NA	Operational Stress subscale from the PSQ<br>Perceived stress<br><br>Mood Disturbance (POMS-SF scale)<br>Depression subscale<br>Confusion subscale<br>Tension subscale<br>Anger subscale	NA

Study	Intervention description	Improved	Worsened	No Change	Qualitative/Self-Report
	techniques such as diaphragmatic breathing.			Fatigue subscale	

Wellness Program

Study	Intervention description	Improved	Worsened	No Change	Qualitative/Self-Report
45	A 6-month, 6-hour comprehensive mind-body wellness intervention focused on healthy eating, exercise, peer support, sleep, stress management, and other lifestyle components and behaviors. The experimental group was organized into small teams (3-7) who then worked the program together and met for 30 minute sessions each month that addressed the above wellness topics.<br><br>Participants responded to surveys at 6, 12, and 24 months.	**6 Months**<br>Fruit consumption<br>Vegetable consumption<br>Fruit / vegetable consumption<br>Healthy eating<br>Sleep quality<br>Sleep quantity<br>General health<br>Stress<br>Tobacco use<br><br>**12 Months**<br>Fruit consumption<br>Fruit / vegetable consumption<br>Healthy eating<br>Alcohol use<br>Systolic blood pressure<br><br>**24 Months**<br>Fruit consumption<br>Vegetable consumption Fruit / vegetable consumption<br>Healthy eating<br>Tobacco use<br><br>**All Follow-ups**<br>Fruit consumption<br>Veg consumption<br>Fruit and vegetable consumption<br>Healthy eating<br>Sleep quality<br>Stress<br>Tobacco use	NA	**6 Months**<br>Physical activity<br>Alcohol use<br>Depression<br><br>**12 Months**<br>Physical activity<br>Sleep quality<br>Sleep quantity<br>General health<br>Stress<br>Tobacco use<br>Depression<br>Diastolic blood pressure<br>BMI<br><br>**24 Months**<br>Vegetable consumption<br>Physical activity<br>Sleep quality<br>Sleep quantity<br>General health<br>Stress<br>Alcohol use<br>Depression<br>Diastolic & systolic blood pressure<br>BMI<br><br>**All Follow-ups**<br>Physical activity<br>Sleep quantity<br>General health<br>Alcohol use<br>Depression<br>Diastolic & systolic blood pressure<br>BMI	In semi-structured group interviews, participants reported:<br><br>- They learned about health and wellness and the benefits of healthy habits (eating well, reduction of tobacco and alcohol use, sleeping well, etc.)<br><br>- Motivation to change seemed to be external<br><br>- Competing life circumstances and responsibilities made it difficult to maintain changes made during the program

Study	Intervention description	Improved	Worsened	No Change	Qualitative/Self-Report
30	A 10-week coping and wellness program for police emergency dispatchers which focused on nutrition, exercise, recognizing stressors, and responding with more favorable behaviors to stress.<br><br>The intervention included an initial 2-hour coping skills seminar, a 1-hour nutrition seminar, and weekly meetings with a performance coach to review nutrition, exercise schedule, and goals for behavioral changes with regard to stress.	Strength<br>Lower body<br>Upper body<br>Avoidance coping strategies<br>Physical energy<br>Stress<br>Trend level improvement (*p* = 0.058) (authors reported as significant)	NA	Approach coping strategies<br>Job satisfaction	NA

Yoga

Study	Intervention description	Improved	Worsened	No Change	Qualitative/Self-Report
31	A 6-week, approximately 6-hour web-based yoga program, with psychoeducation included in modules, based on YogaShield: Yoga for First Responders.	Stress mindset<br>PTSD symptoms<br>Trend level improvement (*p* = 0.05)	NA	Psychological distress<br>Emotional regulation<br>Psychological preparedness<br>Work performance<br>Sickness absence	In a follow-up survey, most participants rated:<br>• The information as easy to find and the modules as useful<br>• Lecture videos, exercises, and yoga videos as helpful<br>• The course as useful, engaging, interesting, and that they would recommend it to a coworker<br>Some participants rated:<br>• The live Zoom classes and discussion forums as unhelpful<br>In an open-ended prompt in the survey asking for feedback about the program, participants reported:<br>• Positively about what was learned in the course<br>• The only major negative aspect was the time needed to complete the program

Study	Intervention description	Improved	Worsened	No Change	Qualitative/Self-Report
46<br><br>Participants were police academy trainees	A 20-week, 7.5 hour Kripalu yoga program for police academy trainees. Kripalu yoga emphasizes breath awareness, postures, relaxation, and meditation. Breathing techniques were taught for stress regulation in real-world situations. Sessions were taught by a retired detective lieutenant of the Massachusetts State Police.	Perceived Stress<br>Mood States global score from POMS-SF<br>Tension / Anxiety subscale<br>Fatigue / Inertia subscale<br>Anger / Hostility subscale<br>Trend level improvement (*p* = 0.07)<br><br>Observing subscale from FFMQ scale<br>Trend level improvement (*p* = 0.10)	NA	Depression / Dejection subscale of POMS-SF<br>Vigor / Activity subscale of POMS-SF<br>Confusion / Bewilderment subscale of POMS-SF<br>FFMQ Mindfulness<br>Describing subscale<br>Awareness subscale<br>Non-judging subscale<br>Non-reacting subscale	Participants rated the three main components of the yoga practice (physical postures, meditation, and breathing exercises) beneficial (Mean range = 5.6-6.4 on a scale from 1-10).<br><br>Responses to open-ended feedback survey:<br><br>• 5 of 24 relay that it was beneficial, relaxing, stress relieving<br><br>• 5 mentioned some resistance because of the topic

Detoxification

Study	Intervention description	Improved	Worsened	No Change	Qualitative/Self-Report
47<br><br>Note that this is a special population of LEOs who experienced symptoms from meth-amphetamine exposure	A 4- to 6-week, 120- to 240-hour detoxification regimen that used daily sauna sessions, nutritional supplementation, and physical exercise.<br><br>The intervention aimed to improve quality of life and chronic health symptoms for LEOs who experienced on-duty exposure to methamphetamine based on evidence that these particular methods can facilitate flushing harmful toxins from the body.	**Short Form Health Survey**<br>Physical function<br>Role limitations due to:<br>Physical health<br>Emotional problems<br>Energy / fatigue<br>Emotional wellbeing<br>Social functioning<br>Pain<br>General health<br>Health from one year ago<br><br>**Symptom Severity & Poor Health Days**<br>Immune system Musculoskeletal<br>GI<br>Cognitive<br>Emotional<br>Neurological<br>Mucus membranes Respiratory<br>Cardiovascular<br>Skin<br><br>**Days of:**<br>Poor physical health<br>Poor mental health<br>Limited activities due to poor health<br>Sick days<br><br>Sleep<br>Neurotoxicity symptoms	NA	**Mini-Mental Status Eval**<br>Cognitive function	While no measures reported any worsening outcomes, a list of some adverse reactions experienced during the protocol is provided:<br><br>• Niacin flush / itchy skin<br>• Emotional, irritable, despondent<br>• Cough, congestion, sore throat<br>• Flu-like symptoms w/ no fever<br>• Flu-like symptoms w/ mild fever<br>• Headache<br>• Sleeplessness, vivid dreams<br>• Fatigue<br>• Stomach cramps, nausea, diarrhea<br>• Body aches<br>• Gout<br>• Work or other schedule conflicts

Sleep

Study	Intervention description	Improved	Worsened	No Change	Qualitative/Self-Report
48	A 12-week sleep-focused program tailored for law enforcement from an existing National Institute for Occupational Safety and Health (NIOSH) program for nurses. After a 2-2.5 hour initial training, participants practiced what was learned about sleep on their own and, beginning at week 11, maintained a sleep diary and wore an actigraph to capture sleep data.	Sleep latency<br>Sleepiness<br>Nightmares<br>Awakenings per hour<br><br>Knowledge about sleep<br><br>Wakefulness during waking hours<br><br>Trouble keeping up enthusiasm for getting things done throughout waking hours	NA	Sleep quantity<br>Sleep quality<br>Use of sleep medication<br>Napping<br><br>Predicted cognitive effectiveness averaged across waking hours<br><br>Self-reported sleep duration<br><br>Awakenings throughout the sleep period<br><br>Disturbance throughout the sleep period from temperature (being too hot or too cold)	In feedback surveys, most participants rated their:<br>- Life has improved<br>- Sleep has improved<br>- Positive behavior change<br>- Improved sleeping environment<br>- Viewing sleep as important<br><br>Feedback surveys from participants indicated that:<br>- Most participants had positive comments about the program<br><br>A little over half of participants had some negative comments:<br>- A lot of reading<br>- Program is too long<br><br>Some suggestions from participants:

Study	Intervention description	Improved	Worsened	No Change	Qualitative/Self-Report
					Audio recorded version of materials to reduce reading time

Note: Mind-body or integrative health interventions performed on law enforcement categorized by type, in descending order of date published.

* This is a composite outcome resulting from principal components analysis combining multiple scales

Supplementary Table 2: Measurement Methods

Self Report	
**Stress, Burnout**	
Burnout	Maslach Burnout Inventory (45)<br>Oldenburg Burnout Inventory (26,33–36,38)
Exhaustion, fatigue	Maastricht Questionnaire 9-item version of Form B (41,42)<br>PROMIS Fatigue (33,34)
Stress	Kessler Psychological Distress Scale, 6-item (31)<br>Operational Police Stress Questionnaire (28,34)<br>Organizational Police Stress Questionnaire (28,34)<br>Perceived Stress Scale (30,33,34,41,42,46)<br>Perceived Stress Scale-4 (26)<br>Perceived Stress Scale-10 (28)<br>Police Stress Questionnaire (26,33,38)<br>Stress Mindset Measure (31)
Stress (including organizational stress)	Personal and Organizational Quality Assessment-R (42)
**Mental Health**	
Anxiety	Hospital Anxiety Scale (39)<br>PROMIS (33,34)

	PROMIS short form, 6 items (38)
Critical Incident Stress & PTSS	Critical Incident History Questionnaire (33)<br>Impact Events Scale (41,42)
Depression	Hospital Depression Scale (39)<br>PROMIS (33,34)<br>PROMIS Depression short form, 6 items (38)
PTSD symptoms	PTSD-8: 8-item abbreviation of the Harvard Trauma Questionnaire (31)<br>PTSD Checklist-Civilian Version (33)<br>PTSD Checklist for DSM-5 (32,34)<br>Sources of Support scale from the National Vietnam Veterans Readjustment Study (49)
Suicidal ideation	7-item Concise Health Risk Tracking scale (38)
**Affect**	
Emotion / affect	Difficulties in Emotion Regulation Scale (26)<br>Emotional Intelligence Scale (26)<br>Emotion Regulation Questionnaire (31)<br>Profile of Mood States-Short Form (28,46)<br>Positive and Negative Affect Schedule (33)
**Physical Health**	
General health	PROMIS Global Health Short-Form (26)<br>Short Form 36, SF-36 (54*)
Pain / physical discomfort	Cornell Musculo-skeletal Discomfort Questionnaire (18 of the 54 items) (54*)

	PROMIS (33,34)
Physical energy	Measure was created by researchers for this study (30)
Physical functioning	Foundation for Advancements in Science and Education (FASE) for clinical settings using the Hubbard regimen (47)<br>PROMIS (33,34)
Neurotoxicity	Questionnaire based on the parameters of Singer (47)
**Job Satisfaction, Performance**	
Job satisfaction	Job Satisfaction measure by Pond and Geyer, 1991 (30)
Work performance	Health and Work Performance Questionnaire (31)
Work Limitations	Work Limitations Questionnaire 34<br>Work Limitations Questionnaire, short form (33)
**Anger and Aggression**	
Aggression	Buss-Perry Aggression Questionnaire-Short Form (37,38)
Anger	PROMIS® (v1.0) short form version of anger, 5 items (38)
**Sleep**	
Sleep	Karolinska Sleepiness Scale (54*)<br>Pittsburgh Sleep Quality Index (32–34,48, 54*)<br>PROMIS short form, 6 items (38)<br>PROMIS sleep (33,34, 54*)<br>Sleep Activity Diary (48)
Sleepiness	Epworth Sleepiness Scale (48)
Sleep knowledge	Sleep knowledge test (48)

Positive Psychology	
Coping strategies	Brief COPE (39)<br><br>Untitled measure: “Participants rated how they typically deal with work-related stressful events. They were first instructed to think of a highly stressful event on the job from recent weeks or months. Then, they rated 24 items, using a 5-point scale (0 = not at all like me, 4 = always like me) on how they responded to the event” (30)
Mindfulness	Five Facet Mindfulness Questionnaire (46)<br><br>Five Facet Mindfulness Questionnaire-Short Form (26,36,38)<br><br>Mindfulness Process Questionnaire (26)
Psychological flexibility	Acceptance and Action Questionnaire-II (36,38)
Psychological preparedness	Psychological preparedness: “...measured using a modified version of a previously validated subscale of psychological preparedness for natural disasters” (31)
Quality of Life	RAND 36-item Short Form Health Survey (47)
Resilience: Cognitive flexibility, meaning-making, active coping, spirituality	Brief Resilience Scale (26)<br><br>Connor-Davidson Resilience Scale (38)<br><br>Personal and Organizational Quality Assessment (41,43,44)<br><br>Response to Stressful Experiences Scale (41,42)
Self-compassion	Self-Compassion Scale-Short Form (36,38)
Wellbeing	Psychological Well-Being Scale, 18 item (33)
**Cognitive**	
Cognitive function	Mini-Mental State Examination (47)
**Behavior**	
Alcohol use	Alcohol Use Disorders Identification Test (34,39)<br><br>Patient Reported Outcomes Measurement Information System (PROMIS; v1.0) short form, 7-item (35,38)

	PROMIS® (v1.0) Alcohol Use-Short Form (36)
Health behaviors	Health Behaviors Checklist (34)
Nutrition	Cancer Institute's fruit and vegetable all day screener (54*)
Stress, healthy eating, and physical activity	No formally validated scale; "Constructs with established reliability from our previous studies were used" (54*)
**Social**	
Social functioning	PROMIS (33,34)
Family functioning	Family Assessment Device (26)
Social support	10-item Sources of Support scale from the National Vietnam Veterans Readjustment Study (39)
**Other**	
Daily report form	A structured summary of vital signs/events recorded by trained staff on each treatment day, including any undesirable effects (whether or not related to treatment) (47)
Expectancy and credibility	Expectancy/Credibility Questionnaire (38)
Feasibility, efficacy, participant feedback	Acceptability: Short survey to determine participants' satisfaction with training program (31)<br>Anonymous survey at the end of intervention (39)<br>Interviews (44)<br>iPod Touch equipped with iMINDr app which has guided practices and monitoring software to track use (38)<br>Number of hours spent working on the program (31)<br>Mentor reports (43)<br>Program evaluation questionnaire (46)<br>Qualitative interviews 6 months post intervention (33)<br>Usability: Short survey to determine level of practical usefulness of training program (31)

Participants' frequency and duration of mindfulness practice	iPod Touch with iMINDr app (37)
**Physical/Physiological Measures**	
Blood pressure	(33,42,44,45)
BMI	Height and weight (42,45)
Cardiovascular disease risk factors	C-reactive protein (CRP) (33,42)<br>Hemoglobin A1c (HbA1c)<br>Low-density lipoprotein-cholesterol (LDL-C)<br>Non-fasting blood sample<br>Total cholesterol<br>(42)
Cortisol, cortisol awakening response, stress reactivity	Hair cortisol (34)<br><br>Salivary cortisol; passive drool method (26,34,38)
Fasting lipid and lipoprotein	(45)
Glucose levels	(45)
Heart rate	FitBit (34,40)<br>Earlobe sensor (41)<br>Pulse reading (33,42)
Heart rate variability (HRV)	Earlobe sensor and HeartMath Inner Balance application for iPad (41)<br><br>Electrocardiogram monitor (42,44)
Height and weight	(33,45)
Hip-to-waist ratio	(33)
Inflammatory markers	Capillary blood on filter paper following lancet prick (34)
Parasympathetic activity	Interbeat intervals (42)
Sleep	Actigraphy (ReadiBand) (48)

	FitBit Charge 2 (32,34)
Strength	Bench press (30)<br>Leg press (30)

*Measures reported in 52 (not included in this review) and cited in 45.

Supplementary Table 3: Organized by Outcomes of Interest

Stress and Burnout (Cortisol in Physiology section)

Study	Improved	Worsened	No Change
31	Stress mindset	NA	NA
40	**Qualitative Data Only**<br>In interviews, participants reported:<br>• Improved stress awareness<br>• Participants preferred graphic heart rate monitor over stress graphic<br>• Reduction in acute stress from breathing exercises	NA	NA
33	Organizational stress subscale from PSQ<br>T2-T1: *p* = 0.054<br>T3-T1: *p* = 0.00042<br>(Note: Group*time not reported. These values are not included in the published paper—authors provided this information upon request.)<br><br>Operational stress subscale from PSQ<br>T2-T1: *p* = 0.03<br>T3-T1: *p* = 0.0016<br>(Note: Group*time not reported. These values are not included in the published paper—authors provided this information upon request.)<br><br>Exhaustion subscale of Burnout measure	NA	Burnout (total measure showed no change)<br>Disengagement (subscale)
34	**Group*time**<br>Distress Component*<br><br>Fatigue	Dose-response<br>More engagement with practice was associated with significantly **increased** occupational stress	**Group*time**<br>Occupational Stress Component*<br><br>Perceived stress

Study	Improved	Worsened	No Change
	OLBI Exhaustion<br><br>**Post Training**<br>Distress Component*<br><br>Fatigue<br>OLBI Exhaustion<br><br>**3 Months**<br>Distress Component*<br><br>Perceived stress<br>Fatigue<br>OLBI Exhaustion		Operational Stress subscale of PSQ<br>Organizational Stress subscale of PSQ<br><br>**Post Training**<br>Occupational Stress Component*<br><br>Perceived stress<br>Operational Stress subscale of PSQ<br>Organizational Stress subscale of PSQ<br><br>**3 Months**<br>Occupational Stress Component*<br><br>Operational Stress subscale of PSQ<br>Organizational Stress subscale of PSQ
35<br><br>Secondary analysis of 38	Burnout	NA	NA
36<br><br>Secondary analysis of 38	Burnout<br><br>Burnout (predicted by increased self-compassion)	NA	NA
38	**Group*time**<br>Burnout<br>Organizational Stress subscale of PSQ<br><br>**Post Analyses**<br>Burnout<br>Organizational Stress subscale of PSQ<br><br>**3 Month Follow-up**<br>NA	NA	**Group*time**<br>Operational Stress subscale of PSQ<br><br>**Post Analyses**<br>Operational Stress subscale of PSQ<br><br>**3 Month Follow-up**<br>Burnout<br>Organizational Stress subscale of PSQ<br>Operational Stress subscale of PSQ
41<br><br>Participants	NA	NA	Perceived stress<br>Vital exhaustion<br>Response to Stressful Experiences

Study	Improved	Worsened	No Change
were police academy recruits			Organizational Stress<br>Pressures of Life<br>Relational Tension<br>Intention to Quit (also listed under Job Satisfaction and Performance)<br>Physical Stress (self-reported measure)<br>Fatigue<br>Health Symptoms
26	Burnout<br>General stress<br>Organizational Stress subscale of PSQ<br>Operational Stress subscale of PSQ	NA	NA
45	**6 Months**<br>Stress<br><br>**12 Months**<br>NA<br><br>**24 Months**<br>NA<br><br>**All Follow-ups**<br>Stress	NA	**6 Months**<br>NA<br><br>**12 Months**<br>Stress<br><br>**24 Months**<br>Stress<br><br>**All Follow-ups**<br>NA
42	No Change in Quantitative Outcomes<br><br>**Qualitative Data**<br>In a survey at the end of the study, participants reported:<br><br>• Improved awareness of stress levels<br><br>• Biofeedback device helped relaxation	NA	Perceived stress<br>Vital exhaustion<br>Response to Stressful Experiences<br><br>Organizational Stress<br>Pressures of Life<br>Relational Tension<br>NS but authors report effect sizes opposite to improvement (*d* = 0.21)<br>Intention to Quit (also listed under Job Satisfaction and Performance)<br>How Stressed Have You Been<br><br>Physical stress (self-reported symptoms)

Study	Improved	Worsened	No Change
			Fatigue<br>Health Symptoms
43	Physical Stress (overall primary scale score)<br>Health Symptoms (subscale)	NA	Organizational Stress (overall primary scale)<br>Pressures of Life (subscale<br>Relational Tension (subscale)<br>Stress (subscale)<br>Intention to Quit (also listed under Job Satisfaction and Performance)<br>Fatigue (subscale of Physical Stress)
46<br><br>Participants were police academy trainees	Perceived stress	NA	NA
30	Stress<br>Trend improvement ($p = 0.058$) (Note: Authors reported as significant)	NA	NA
44	**Qualitative Data**<br>In comments during the semi-structured interviews that measured work performance, coping skills, family relationships, and interpersonal skills, the experimental group indicated that, overall:<br><br>• Awareness of their stress and emotions improved<br><br>• Training improved participants' capacity to handle stress	NA	Fatigue
28	Organizational stress	NA	Operational stress<br>Perceived stress

Mental Health

Study	Improved	Worsened	No Change
32<br><br>Secondary analysis of 34	PTSD symptoms	NA	NA
31	PTSD symptoms<br>Trend level improvement (*p* = 0.05)	NA	Psychological distress<br>Emotional regulation
33	PTSD symptoms<br>Anxiety<br>Negative affect<br>Depression<br>Trend level improvement (*p* = 0.06)<br>(Note: Authors table indicates *p* = 0.067 whereas results report 0.060)	NA	Positive affect
34	**Group*time**<br>Distress Component* (also listed under Stress and Burnout)<br><br>PTSD Checklist<br>Anxiety<br><br>**Post Training**<br>Distress Component* (also listed under Stress and Burnout)<br><br>PTSD Checklist<br>Anxiety<br><br>**3 Months**<br>Distress Component* (also listed under Stress and Burnout)<br><br>Anxiety<br>Depression	NA	**Group*time**<br>Depression<br><br>**Post Training**<br>Depression<br><br>**3 Months**<br>PTSD Checklist
38	NA	NA	**Group*time**<br>Anxiety

Study	Improved	Worsened	No Change
			Depression<br>Suicidal ideation<br><br>**Post Analyses**<br>Anxiety<br>Depression<br>Suicidal ideation<br><br>**3 Month Follow-up**<br>Anxiety<br>Depression<br>Suicidal ideation
39<br><br>Participants were police academy trainees	Anxiety	NA	PTSD<br>Depression
41<br><br>Participants were police academy recruits	NA	NA	Impact of Events Scale<br>Intrusive subscale<br>Avoidance subscale<br>Emotional Vitality<br>Emotional Buoyancy<br>Emotional Contentment<br>Emotional Stress<br>Anxiety and Depression
26	Mental health<br>Difficulties with emotion regulation	NA	NA
45	NA	NA	**6 Months**<br>Depression<br><br>**12 Months**<br>Depression<br><br>**24 Months**<br>Depression

Study	Improved	Worsened	No Change
			**All Follow-ups**<br>Depression
42	NA	NA	Impact of Events Scale<br>Intrusive subscale<br>NS but authors report effect sizes opposite to improvement (*d* = 0.26)<br>Avoidance subscale<br>NS but authors report effect sizes opposite to improvement (*d* = 0.29)<br>Emotional Vitality<br>Emotional Buoyancy<br>Emotional Contentment<br>Emotional Stress<br>Anxiety and Depression
43	Emotional Vitality (primary scale)<br>Emotional Contentment (subscale)	NA	Emotional Stress<br>Anxiety & Depression<br>Emotional Buoyancy (subscale of Emotional Vitality primary scale of the POQA)
46<br><br>Participants were police academy trainees	Mood states global score (global score of POMS-SF also showed improvement)<br>Tension / Anxiety<br>Fatigue / Inertia	NA	Depression / Dejection subscale of POMS-SF<br>Vigor / Activity subscale of POMS-SF<br>Confusion / Bewilderment subscale of POMS-SF
44	Distress<br>Global negative emotions<br>Depression<br><br>**Qualitative Data**<br>In comments during the semi-structured interviews that measured work performance, coping skills, family relationships, and interpersonal skills, the experimental group indicated that, overall:<br><br>- Training was beneficial to managing mood	NA	Sadness<br>Positive emotion<br>Anxiety

Study	Improved	Worsened	No Change
28	NA	NA	Mood<br>Mood disturbance<br>Depression<br>Confusion<br>Tension<br>Fatigue
47<br><br>**Note that this is a special population of LEOs who experienced symptoms from methamphetamine exposure**	**Short Form Health Survey**<br>Role limitations due to:<br>Emotional problems<br>Emotional wellbeing<br><br>**Symptom Severity & Poor Health Days**<br>Emotional<br><br>Days of:<br>Poor mental health	NA	**Mini-Mental Status Eval**<br>NA

Job Satisfaction and Performance

Study	Improved	Worsened	No Change
31	NA	NA	Work performance<br>Sickness absence
33	NA	NA	Health-related productivity loss
34	**Group*time**<br>NA<br><br>**Post Training**<br>NA<br><br>**3 Months**<br>NA	NA	**Group*time**<br>Work limitations<br><br>**Post Training**<br>Work limitations<br><br>**3 Months**<br>Work limitations
41	NA	NA	Intention to Quit subscale of the Organizational Stress primary scale of the POQA (also listed under Stress and Burnout)
42	NA	NA	Intention to Quit subscale of the Organizational Stress primary scale of the POQA (also listed under Stress and Burnout)
43	NA	NA	Intention to Quit subscale of the Organizational Stress primary scale of the POQA (also listed under Stress and Burnout)
30	NA	NA	Job satisfaction
44	Work Performance self-ratings from a four-week post-interview of Program Impact Assessment	NA	Job satisfaction<br>Productivity
47<br><br>**Note that this is a special population of LEOs who experienced symptoms from**	**Short Form Health Survey**<br>Role limitations due to:<br>Physical health<br>Emotional problems<br><br>**Symptom Severity & Poor Health Days**<br>NA<br><br>Days of:	NA	NA

Study	Improved	Worsened	No Change
**methamphetamine exposure**	Limited activities due to poor health<br>Sick days		

Anger and Aggression

Study	Improved	Worsened	No Change
37<br><br>Secondary analysis of 38	Aggression<br><br>(Frequency and duration of mindfulness practices was significantly correlated with reduced aggression)	NA	NA
38	**Group*time:**<br>Aggression<br><br>**Post Analyses**<br>Aggression<br><br>**3 Month Follow-up**<br>NA	NA	**Group*time**<br>Anger<br><br>**Post Analyses**<br>Anger<br><br>**3 Month Follow-up**<br>Anger<br>Aggression
41<br><br>Participants were police academy recruits	NA	NA	Anger and Resentment subscale of the Emotional Stress primary scale of the POQA
26	Anger	NA	NA
42	NA	NA	Anger and Resentment subscale of the Emotional Stress primary scale of the POQA
43	NA	NA	Anger and resentment subscale of the Emotional Stress primary scale of the POQA
46<br><br>Participants were police academy trainees	Anger / Hostility subscale of POMS-SF<br>Trend level improvement (p = 0.07)	NA	NA

Study	Improved	Worsened	No Change
44	NA	NA	Anger
28	NA	NA	Anger

Sleep

Study	Improved	Worsened	No Change
32<br><br>Secondary analysis of 34	Subjective sleep quality (Subjective sleep quality improvement was mediated by reductions in PTSD symptoms)	NA	Objective sleep measures (duration & efficiency)
48	Sleep latency<br>Sleepiness<br>Nightmares<br>Awakenings per hour<br><br>Knowledge about sleep<br><br>Wakefulness during waking hours<br><br>Trouble keeping up enthusiasm for getting things done throughout waking hours	NA	Sleep quantity<br>Sleep quality<br>Use of sleep medication<br>Napping<br><br>Predicted cognitive effectiveness averaged across waking hours<br><br>Self-reported sleep duration<br><br>Awakenings throughout the sleep period<br><br>Disturbance throughout the sleep period from temperature (being too hot or too cold)
33	Sleep quality	NA	Sleep disturbances
34	**Group*time**<br>Sleep Component*<br>PROMIS sleep disturbances<br><br>**Post Training**<br>PROMIS sleep disturbances<br><br>**3 Months**<br>Sleep Component*<br>Pittsburgh sleep<br>PROMIS sleep disturbances	NA	**Group*time**<br>Pittsburgh sleep<br><br>**Post Training**<br>Sleep Component*<br>Pittsburgh sleep<br><br>**3 Months**<br>NA
38	**Group*time**<br>Sleep disturbance<br><br>**Post Analyses**<br>Sleep disturbance	NA	**Group*time**<br>NA<br><br>**Post Analyses**<br>NA

Study	Improved	Worsened	No Change
	Trend level improvement ($p = 0.08$)<br><br>**3 Month Follow-up**<br>NA		**3 Month Follow-up**<br>NA
39<br><br>Participants were police academy trainees	NA	NA	Sleep disruption
45	**6 Months**<br>Sleep quality<br>Sleep quantity<br><br>**12 Months**<br>NA<br><br>**24 Months**<br>NA<br><br>**All Follow-ups**<br>Sleep quality	NA	**6 Months**<br>NA<br><br>**12 Months**<br>Sleep quality<br>Sleep quantity<br><br>**24 Months**<br>Sleep quality<br>Sleep quantity<br><br>**All Follow-ups**<br>Sleep quantity
44	NA	NA	Sleeplessness

Positive Psychology

Study	Improved	Worsened	No Change
31	NA	NA	Psychological preparedness
33	Wellbeing	NA	NA
36<br><br>Secondary analysis of 38	Pre-post intervention improvements in mindfulness predicted lower problematic alcohol use (also listed under Health Behaviors)	NA	Non-significant improvements in self-compassion predicted post-intervention burnout<br><br>Neither pre-post intervention improvements in self-compassion nor psychological flexibility predicted decreased problematic alcohol use<br><br>Neither pre-post intervention changes in mindfulness nor psychological flexibility predicted reductions in burnout
38	**Group*time**<br>Non-reactivity subscale from FFMQ<br>Psychological flexibility<br><br>**Post Analyses**<br>Non-reactivity subscale from FFMQ<br>Psychological flexibility<br><br>**3 Month Follow-up**<br>NA	NA	**Group*time**<br>FFMQ<br>Non-judging subscale<br>Acting with Awareness subscale<br>Resilience<br>Self-compassion<br><br>**Post Analyses**<br>FFMQ<br>Non-judging subscale<br>Acting with Awareness subscale<br>Resilience<br>Self-compassion<br><br>**3 Month Follow-up**<br>FFMQ<br>Non-judging subscale<br>Acting with Awareness subscale<br>Non-reactivity subscale<br>Psychological flexibility<br>Resilience<br>Self-compassion
26	Mindful process	NA	NA

Study	Improved	Worsened	No Change
	Mindfulness<br>Non-reactivity<br>Non-judging<br>Act with awareness<br>Resilience		
46<br><br>Participants were police academy trainees	Observing subscale of FFMQ scale<br>Trend level improvement ($p = 0.10$)	NA	Mindfulness (FFMQ)<br>Describing subscale<br>Awareness subscale<br>Non-judging subscale<br>Non-reacting subscale
44	Vitality<br>Trend level improvement ($p < 0.056$) (Note: The publication had *p* values which differed and the value of 0.056 was confirmed by authors via email.)<br>Peacefulness<br>Trend level improvement ($p < 0.056$) (Note: The publication had *p* values which differed and the value of 0.056 was confirmed by authors via email.)<br><br>**Qualitative Data**<br>In comments during the semi-structured interviews that measured work performance, coping skills, family relationships, and interpersonal skills, the experimental group indicated that, overall:<br><br>Awareness of their stress and emotions improved	NA	NA

Cognitive

Study	Improved	Worsened	No Change
48	NA	NA	Predicted cognitive effectiveness averaged across waking hours

Study	Improved	Worsened	No Change
39<br><br>Participants were police academy trainees	Coping strategies of:<br>Positive reframing<br>Humor	NA	Coping strategies of:<br>Active coping<br>Positive planning<br>Self-blame
26	Emotional intelligence	NA	NA
30	Avoidance coping strategies	NA	Approach coping strategies
44	NA	NA	Coping Skills self-ratings from a four-week post-interview of Program Impact Assessment<br>Mental clarity<br>Goal clarity
47<br><br>**Note that this is a special population of LEOs who experienced symptoms from methamphetamine exposure**	**Short Form Health Survey**<br>NA<br><br>**Symptom Severity & Poor Health Days**<br>Cognitive	NA<br>NA	**Mini-Mental Status Eval**<br>Cognitive function

Health Behaviors

Study	Improved	Worsened	Stayed the same
35<br><br>Secondary analysis of 38	Pre-post reductions in problematic alcohol use over time were mediated by reduced burnout	NA	NA

Study	Improved	Worsened	Stayed the same
34	**Group*time**<br>NA<br><br>**Post Training**<br>NA<br><br>**3 Months**<br>NA	NA	**Group*time**<br>Health behavior checklist<br><br>**Post Training**<br>Health behavior checklist<br><br>**3 Months**<br>Health behavior checklist
36<br><br>Secondary analysis of 38	Pre-post intervention improvements in mindfulness predicted lower problematic alcohol use (also listed under Positive Psychology)	NA	Neither pre-post intervention improvements in self-compassion nor psychological flexibility predicted decreased problematic alcohol use
38	**Group*time**<br>Alcohol use<br><br>**Post Analyses**<br>NA<br><br>**3 Month Follow-up**<br>NA	NA	**Group*time**<br>NA<br><br>**Post Analyses**<br>Alcohol use<br><br>**3 Month Follow-up**<br>Alcohol use
39<br><br>Participants were police academy trainees	Alcohol use	NA	NA
45	**6 Months**<br>Fruit consumption<br>Vegetable consumption<br>Fruit / vegetable consumption<br>Healthy eating<br>Tobacco use<br><br>**12 Months**<br>Fruit consumption<br>Fruit / vegetable consumption<br>Healthy eating	NA	**6 Months**<br>Physical activity<br>Alcohol use<br><br>**12 Months**<br>Physical activity<br>Tobacco use<br><br>**24 Months**<br>Vegetable consumption<br>Physical activity

Study	Improved	Worsened	Stayed the same
	Alcohol use<br><br>**24 Months**<br>Fruit consumption<br>Vegetable consumption Fruit / vegetable consumption<br>Healthy eating<br>Tobacco use<br><br>**All Follow-ups**<br>Fruit consumption<br>Veg consumption<br>Fruit and vegetable consumption<br>Healthy eating<br>Tobacco use<br><br>**Qualitative Data**<br>In semi-structured group interviews, participants reported:<br><br>• They learned about health and wellness and the benefits of healthy habits (eating well, reduction of tobacco and alcohol use, sleeping well, etc.)<br><br>• Motivation to change seemed to be external		Alcohol use<br><br>**All Follow-ups**<br>Physical activity<br>Alcohol use<br><br>**Qualitative Data**<br>In semi-structured group interviews, participants reported that competing life circumstances and responsibilities made it difficult to maintain changes made during the program

Social

Study	Improved	Worsened	No Change
33	NA	NA	Social participation<br>NS but effect sizes show numeric worsening (T2-T1 d = 0.28; T3-T1 d = 0.22)
34	**Group*time**<br>NA<br><br>**Post Training**	NA	**Group*time**<br>Social participation<br><br>**Post Training**

Study	Improved	Worsened	No Change
	NA<br><br>**3 Months**<br>NA		Social participation<br><br>**3 Months**<br>Social participation
39<br><br>Participants were police academy trainees	NA	NA	Social support
26	NA	NA	Family functioning
44	**Qualitative Data**<br>In comments during the semi-structured interviews that measured work performance, coping skills, family relationships, and interpersonal skills, the experimental group indicated that, overall:<br><br>- Communication and listening skills were improved	NA	Family Relationships and<br>Interpersonal Skills self-ratings from a four-week post-interview of Program Impact Assessment<br><br>Social support<br>Communication effectiveness
47<br><br>**Note that this is a special population of LEOs who experienced symptoms from methampheta mine exposure**	**Short Form Health Survey**<br>Social functioning<br><br>**Symptom Severity & Poor Health Days**<br>NA	NA	NA

Physical/Physiological

Study	Improved	Worsened	No Change
33	NA	NA	Log-transformed high-sensitivity C-reactive protein<br>Blood pressure<br>Diastolic<br>Systolic<br>Resting pulse rate<br>Pain interference<br>NS but effect sizes show numeric worsening (T2-T1 *d* = 0.35; T3-T1 *d* = 0.42)<br>Pain intensity<br>NS but effect sizes show numeric worsening (T2-T1 *d* = 0.30; T3-T1 *d* = 0.38)<br>Physical functioning<br>NS but effect sizes show numeric worsening (T2-T1 *d* = 0.10; T3-T1 *d* = 0.33)
34	**Group*time**<br>Cortisol Awakening Response (CAR)<br>Trend level improvement (p = 0.06)<br><br>**Post Training**<br>NA<br><br>**3 Months**<br>Dose response for physical health<br>Cortisol Awakening Response (CAR)	NA	**Group*time**<br>Pain Component*<br>Physical Health Component*<br><br>Pain intensity<br>Pain interference<br>Physical function<br><br>Diurnal cortisol slope<br>Hair cortisol concentration<br>Inflammatory markers<br>CRP<br>Cytokines<br><br>**Post Training**<br>Pain Component*<br>Physical Health Component*<br><br>Pain intensity<br>Pain interference<br>Physical function<br><br>Cortisol Awakening Response (CAR)

Study	Improved	Worsened	No Change
			Diurnal cortisol slope<br>Hair cortisol concentration<br>Inflammatory markers<br>CRP<br>Cytokines<br><br>**3 Months**<br>Pain Component*<br>Physical Health Component*<br><br>Pain intensity<br>Pain interference<br>Physical function<br><br>Diurnal cortisol slope<br>Hair cortisol concentration<br>Inflammatory markers<br>CRP<br>Cytokines
38	**Group*time**<br>Cortisol (this result was a group-by-time by day-by-minute)<br><br>**Post Analyses**<br>Cortisol (improvement found on day three post training)<br><br>**3 Month Follow-up**<br>NA	NA	**Group*time**<br>NA<br><br>**Post Analyses**<br>NA<br><br>**3 Month Follow-up**<br>Cortisol
41<br><br>Participants were police academy recruits	Coherence (a state of increased synchronization between higher-order brain activity and the two branches of the autonomic nervous system and a shift toward greater parasympathetic dominance)	NA	NA
26	Physical health<br>Pain interference<br>Trend level improvement (*p* = 0.07)	NA	Salivary cortisol levels

Study	Improved	Worsened	No Change
45	**6 Months**<br>General health<br><br>**12 Months**<br>Systolic BP<br><br>**24 Months**<br>NA<br><br>**All Follow-ups:**<br>NA	NA	**6 Months**<br>NA<br><br>**12 Months**<br>General health<br>Diastolic BP<br>BMI<br><br>**24 Months**<br>General health<br>Diastolic & systolic BP<br>BMI<br><br>**All Follow-ups**<br>General health<br>Diastolic & systolic BP<br>BMI
42	HbA1c<br>Heart rate variability<br>Coherence (Did not include full sample of participants; not everyone returned measuring equipment)	NA	LDL cholesterol<br>**NS** but effect sizes opposite to improvement (d = 0.27)<br>Blood pressure<br>Cholesterol<br>Triglycerides<br>CRP (inflammatory marker)<br>BMI<br>Waist circumference
30	Strength<br>Lower body<br>Upper body<br>Physical energy	NA	NA
44	NA	Rapid heart rate (subjective report)	Body aches<br>Indigestion
47<br><br>Note that this is a special population of	**Short Form Health Survey**<br>Physical function<br>Energy / fatigue<br>Pain<br>General health	NA	NA

Study	Improved	Worsened	No Change
LEOs who experienced symptoms from meth-amphetamine exposure	Health from one year ago<br><br>**Symptom Severity & Poor Health Days**<br>Immune system Musculoskeletal<br>GI<br>Neurological<br>Mucus membranes Respiratory<br>Cardiovascular<br>Skin<br>Neurotoxicity symptoms<br>Days of:<br>Poor physical health<br>Limited activities due to poor health<br>Sick days		

Note: Outcomes of law enforcement mind-body or integrative health interventions categorized by type, in descending order of date published.

* This is a composite outcome resulting from principal components analysis combining multiple scales
